# The SElf-Care After REnal Transplantation Study: A Retrospective Evaluation of a Home-Monitoring Program Implemented as Standard Care

**DOI:** 10.3389/ti.2024.13192

**Published:** 2024-07-22

**Authors:** B. Hezer, M. E. J. Reinders, M. W. F. van den Hoogen, M. Tielen, J. van de Wetering, D. A. Hesselink, E. K. Massey

**Affiliations:** Erasmus MC Transplant Institute, Department of Internal Medicine, University Medical Center Rotterdam, Rotterdam, Netherlands

**Keywords:** kidney transplantation, self-management, telemedicine, eHealth, adherence, self-monitoring

## Abstract

After transplantation self-management is essential for graft survival and optimal quality of life. To address the need for home-based support in self-management, we implemented the “SelfCare after Renal Transplantation” (SeCReT) box, containing home-monitoring equipment combined with a smartphone application that was linked to the electronic patient records. This study investigated the uptake and continuation, protocol adherence, and subjective evaluation of this home-monitoring program. All “*de novo*” kidney recipients who received the SeCReT-box in the study period (Aug 2021–Dec 2022) were eligible for inclusion. Protocol adherence was defined as ≥75%. Subjective evaluation was assessed with a 5-item questionnaire. Of the 297 recipients transplanted, 178 participants (60%) were included in the analysis. Protocol adherence was 83%, 73%, 66%, and 57% respectively at 5, 10, 20, and 40 weeks of the protocol. With regard to continuation, 135 participants were still in the program at the end of the study period (75% retention rate). Regarding subjective evaluations, 82% evaluated the program positively, and 52% reported lower care needs due to home-monitoring. Results are positive among those who entered and continued the program. Qualitative research is needed on barriers to entering the program and facilitators of use in order to promote optimal implementation.

## Graphical Abstract

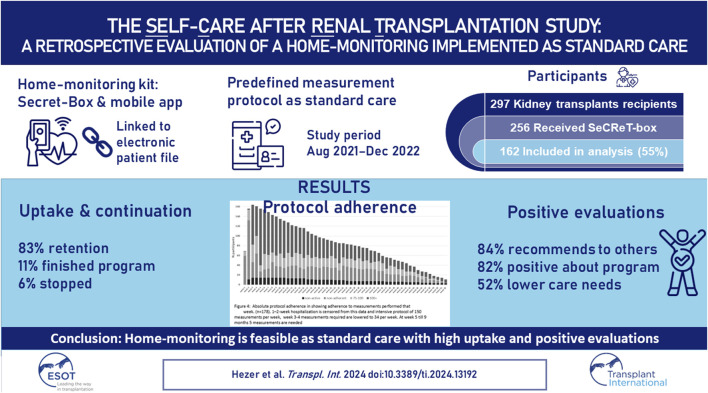

## Introduction

Monitoring physical and lifestyle parameters are essential components of self-management after transplantation. Monitoring has traditionally taken place in the hospital, with measurements carried out by the physician. With increasing technical developments, it has also become feasible for patients to perform reliable medical measurements themselves, outside the healthcare institution. By combining these self-measurements with the use of information and communication technologies, telemedicine becomes possible and is increasingly implemented to support (post-transplant) self-management and care from a distance. Telemedicine is the use of electronic devices to provide medical care from a distance, including teleconsultations and home-monitoring of clinical parameters. Innovations in telemedicine are developing rapidly in the field of kidney transplantation, accelerated by the COVID-19 pandemic. Publications prior to COVID-19 already highlighted the potential benefits of telemedicine in transplantation. There was some evidence that telemedicine was feasible and acceptable among kidney transplant recipients (KTRs) [1, 2], however, the number of studies among adult KTRs was limited and sample sizes were often small [3, 4]. Implementation of home-monitoring after transplantation has been hindered by barriers such as low eHealth literacy [5], availability of equipment, reimbursement of costs and accessibility to internet [6, 7]. Another factor that may negatively influence patient satisfaction and ultimately engagement with the system is the burden associated with carrying out the home-monitoring. However, adherence to home-monitoring protocols and associated satisfaction has yet to be investigated. Potential benefits of being able to monitor vital post-transplant parameters at home include increased accuracy of measurements conducted [8] and improved self-management and disease understanding [9]. From an economic perspective telemedicine also has the potential to reduce costs for patients [2] and for (medium and high volume) transplant centers [10].

Plans to implement home-monitoring in our center were triggered by the COVID-19 pandemic during which KTRs were at increased risk of morbidity and mortality [11] due to their suppressed immune system and poorer response to vaccination compared to controls [12]. Given this heightened risk and social distancing recommendations, hospital-based post-transplant monitoring and care became a challenge [13–15]. To address the need for home-based monitoring and treatment, we developed and implemented the “SelfCare after Renal Transplantation” (SeCReT) box containing home-monitoring equipment combined with a smartphone application that was linked to the electronic patient records. This home-monitoring system has since been adopted as standard care for all KTRs at our transplant center. This study is the first evaluation of this home-monitoring system and aims to evaluate uptake and continuation, adherence to the measurement protocol, subjective evaluation and the relationship between the latter two. Findings can provide targets for future improvement of the system.

## Materials and Methods

### Participants

Patients above 18 years who receive a kidney transplant at our center all receive a SeCReT-box as standard. Recipients who followed the “do novo kidney transplant” home-monitoring protocol during the study period (Aug 2021–Dec 2022) were included in this analysis. Exclusion criteria were insufficient understanding of Dutch or English, following an alternative home-monitoring protocol, more than 4 weeks between transplantation and registration and imminent transfer to another hospital.

### Home-Monitoring System

#### Secret-Box

The SeCReT-box contains the following medically certified devices: a thermometer (either Braun, Kronenbreg, Germany; IRT6520 (Thermoscan 7) or Braun IRT3030), pulse-oximeter (iHealth Air Pulse Oximeter PO3M, San Jose, California, United States), optional weighing scale (iHealth Lina Smart Scale) and blood pressure monitor (iHealth Track Blood Pressure Monitor KNT550BT) with Bluetooth capabilities.

#### Luscii^®^ Application

**FIGURE 1:**
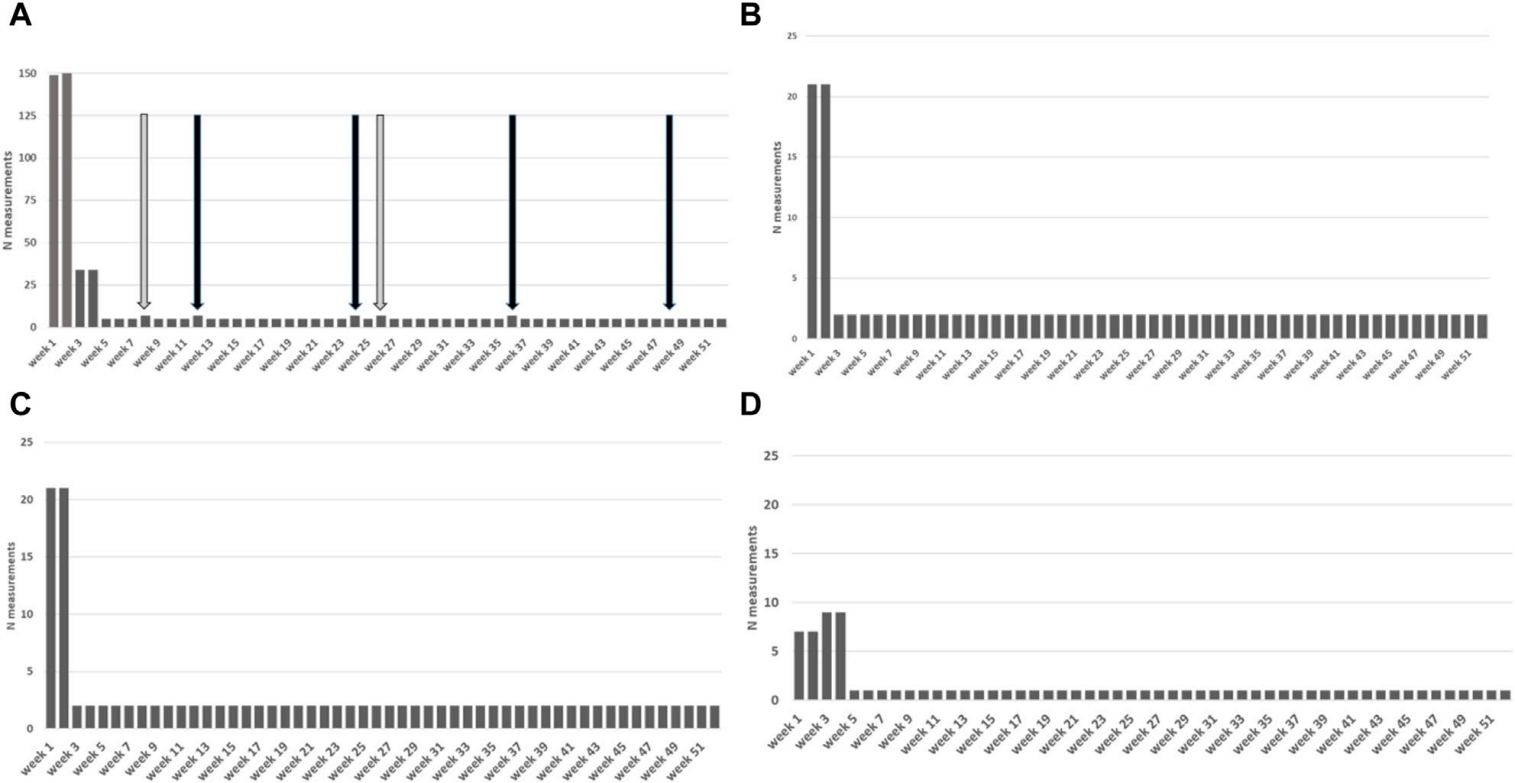
Total number of measurements stipulated by the protocol per week for the first year. **(A)** Total measurements. Black arrows: subjective evaluations/medication; Grey arrows: smoking/sex surveys. **(B)** Heart rate measurements **(C)** Blood pressure measurements **(D)** Weight measurements.

**FIGURE 2:**
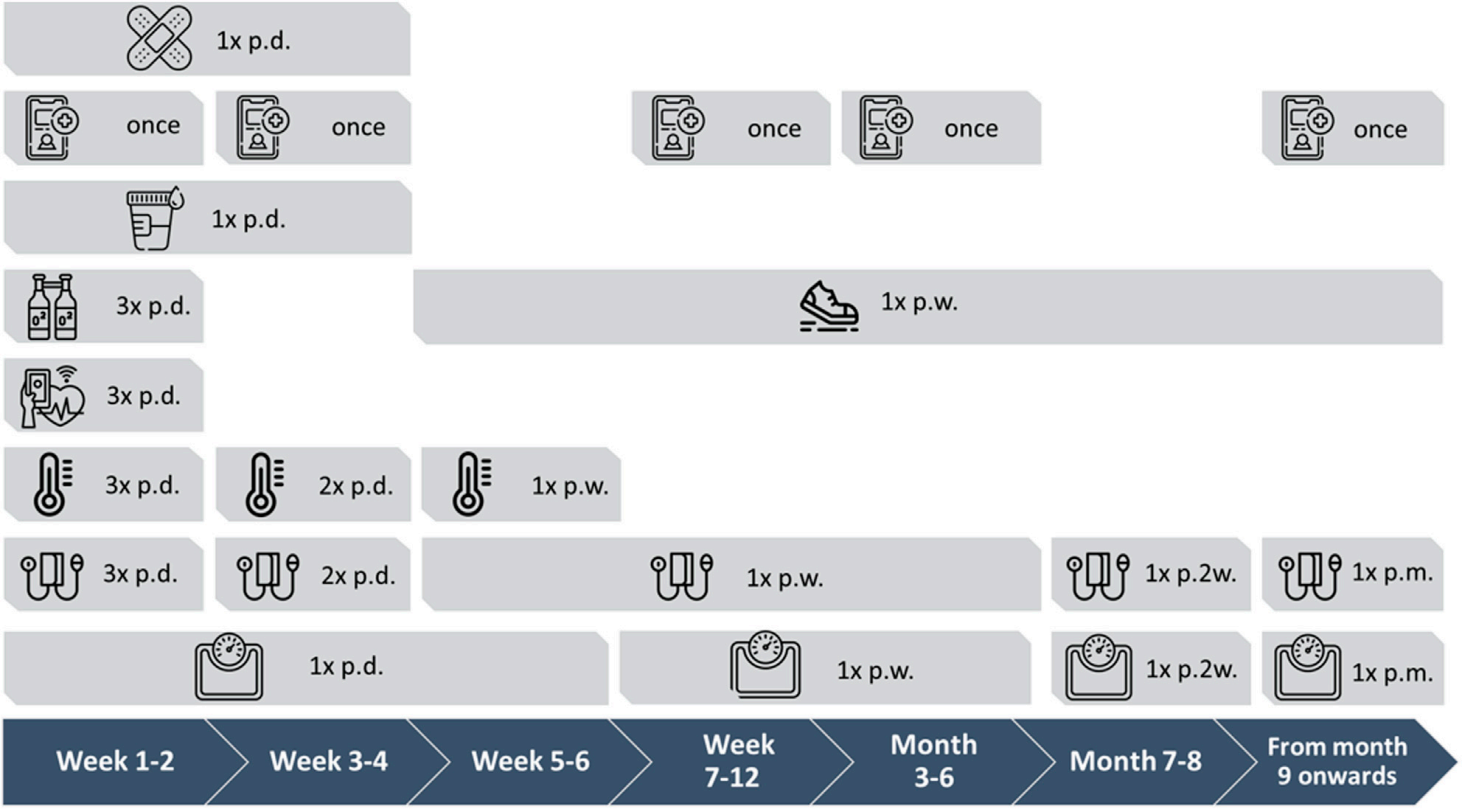
Measurement protocol visualized over 1 year period with measurement frequencies. Frequencies: p.d. = per day, 1x = single time, p.w. = per week, p.2w. = per 2 weeks, p.m. = per month Icons: Band aid = wound checkup; telephone with plus icon = surveys (sex, smoking, subjective evaluation); urine cup = urine and intake liquid volumes; O_2_ bottles = oxygen saturation; shoes = number of steps; heart = heart rate; thermometer = temperature; blood pressure monitor = blood pressure; weighing scale = weight.

Devices in the SeCReT-box are used to measure vital parameters and data is entered by patients manually into the Luscii^®^ smartphone and tablet application (Utrecht, Netherlands). Data from the Luscii^®^ application is integrated into the electronic patient records so that professionals can view and discuss the data during consultations in the outpatient clinic. A kidney transplant-specific home-monitoring protocol was developed in Luscii^®^ that allows data entry from the SeCReT-box, provides information about kidney transplantation, collects data via questionnaires and provides a dashboard for recipients to enter measurements from the various devices. Participants were required to enter measurements such as heart rate, blood pressure and temperature as well as answer survey questions. Frequency of measurements per parameter was predefined for the first 12 months post-transplant and an overview was available for patients in the app homescreen, see Figure 1. Vital signs such as heart rate, blood pressure and temperature were asked frequently (daily/twice weekly/monthly) while other symptoms such as smoking were asked at specific intervals. Intensity of the protocol decreased over time (see Figure 2 and Annex of the Supplementary Material). The app produces a notification signal when a measurement should be taken. If values entered were outside the target value, patients received an alert with instructions on the required action (e.g., contact the out-patient clinic or other). In the app, information was included on how to use the app, how to perform measurements correctly at home, useful websites, advice on living with kidney transplant, medication use and side effects, nutritional advice, sex after transplantation, mental health support sites and how and when to contact the hospital. For technical support we had different lines to communicate: A physical location for SeCReT-box related problems; via Luscii^®^ communication for app-related and our staff to guide patients. The holiday mode in the app allowed for a pause in home-monitoring protocol in the case of holiday or short hospital admissions. For longer admissions, patients were transferred to an alternative protocol and were not included in this analysis.

### Procedure

Recipients were offered the SeCReT-box and the access to the smart-phone application as soon as possible in the first week after transplantation during the hospital admission, free of charge and as part of standard care. The patient was required to have their own smart telephone or tablet. During hospital admission professionals gave tutorials individually to the patient on how to use the devices and Luscii application in order to familiarize them with the process and give them the opportunity to ask questions if needed. Data was collected after following the tutorial during hospitalization. For this study we include data from the day of discharge, once the patient’s is back home. Data from the Luscii^®^ application used in this analysis were extracted on 31-12-2022 and combined with data from the medical records. The study protocol was approved by Institutional Review Board (IRB) of our transplant center (MEC-2023-0143).

### Measures

#### Participant Characteristics

The following socio-demographic characteristics and medical variables were collected from the medical records: age (years), gender (M/F), number of kidney transplants, and date of transplant.

#### Uptake and Continuation

Luscii^®^ records the date of registration, date of activation and date of deactivation. These time-points were used to assess frequency of uptake and continuation. To measure uptake, we define “active users” as those who registered for the home-monitoring system, who activated the app and entered at least one measurement. “Non-active users” were those who registered but did not activate the app or make a minimum of one measurement. Continuation was defined as active participation (entry of measurements) up to the moment of data extraction from the app. The protocol is pre-defined for 12 months until the KTR is referred to a regional hospital who will take over the care of the transplant (end of program). Currently, use of the SeCReT-box and Luscii app is not transferred with the participant to the regional hospital.

#### Protocol Adherence

Protocol non-adherence was defined as <75% of protocolled measurements required that week or overall. To determine total weekly protocol adherence, we summed the number of measurements over all parameters and compared this to the number of measurements stipulated by the protocol for each week that the participant was in the program. All protocol measurements were clustered within each week (7 days) from date of discharge (end of run-in period). We presented the total weekly protocol adherence as a percentage: participants who on average scored <75%, 75%–100%, and ≥100%. For protocol adherence per parameter the number of measurements carried out over the study-period were compared to the number of measurements stipulated by the protocol.

#### Subjective Evaluation

Experience with the home-monitoring system was explored using 5 items provided by Luscii^®^ that were rated on a Likert scale. The specific questions were: What do you think of the remote care service in general (1 – I completely dislike this type of care service; 5 – I think this type of care service is fantastic); Remote monitoring with this app makes me feel safe (1 – strongly disagree; 5 - strongly agree); Thanks to this way of remote monitoring I don’t have to go the hospital or GP as often (1 – strongly disagree; 5 - strongly agree); Remote monitoring with this app improves my insight in my medical condition (1 – strongly disagree; 5 - strongly agree); and How likely is it that you would recommend remote care using this app to other patients? (0- completely unlikely, 10 – very likely). The Likert 5-scale was converted into three categories: negative (score = 1-2), neutral (score = 3), and positive (score = 4-5). The Likert 10-scale was converted into the same three categories: negative (score = 1–4, neutral (score = 5-6), positive (score = 7–10). Each item was analyzed individually. Multiple responses were averaged per participant and first and last scores were compared to assess change over time.

### Statistical Analysis

SPSS version 22 (IBM) was used to analyze the data. Student t-tests were used to compare means between active and non-active users. Frequencies were explored to assess uptake and continuation. In line with the intention to treat principle both groups active and non-active were included in analyses of protocol adherence. Protocol adherence was calculated per week according to the proportion of participants adherent and non-adherent. Mean subjective evaluations were calculated per item. First and last evaluations were compared using a paired t-test for the 50 users with multiple evaluations. Subjective evaluation items were analyzed using a within group linear model to assess if subjective evaluation changed over time. The level of protocol adherence was compared between participants who evaluated the app as positive, neutral, negative or did not give an evaluation. These groups were compared using one-way ANOVA.

## Results

**FIGURE 3:**
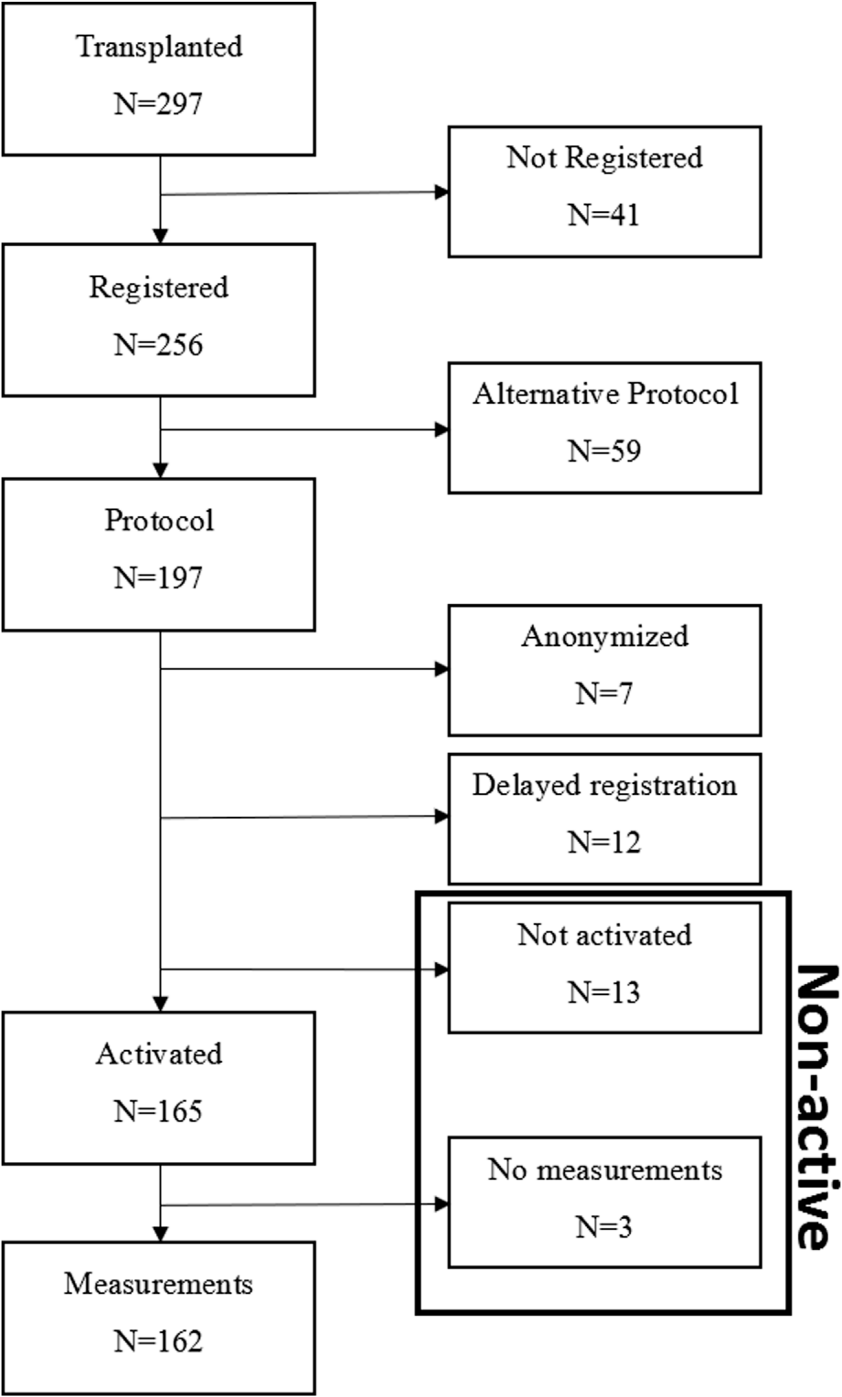
Flow-chart of inclusion. Black box indicates the non-active user group who are included according to the intention to treat principle.

During the study period 297 recipients received a kidney transplantation (see Figure 3). Of these, 256 were registered for home-monitoring; 41 recipients were not registered (no registration of reasons). Of the 256 registered, 59 recipients used the home-monitoring system according to an alternative protocol (blank protocol without predetermined measurement schedule and/or a COVID protocol), resulting from COVID-19 infection or hospitalization. These KTRs were not included in this analysis. Of the 197 participants who registered for the “*de novo* kidney transplant” protocol, 7 recipients requested their data to be anonymous in Luscii^®^, 12 participants were transplanted and registered during the inclusion period but entered the program more than 4 weeks after transplantation, 13 participants did not activate the app and 3 participants did not record any measurements after activation. 162 participants did record measurements out of this group 6 recipients were still in the run-in period after transplantation.

### Uptake and Continuation

Table 1 presents demographic and medical characteristics of the registered participants who were divided into two groups: active users (minimum 1 measurement registered; n = 162) and non-active users (non-activated participants and no-measurements performed participants; n = 16) (Figure 3). Active users were significantly younger than non-active users (*p* = 0.002). Among active users the age ranged from 20 to 82 years with median of 55; and among non-active users the age ranged from 46 to 79 years with median of 67. In the active user group, there was a higher proportion in patients who had received a kidney from a living donor than in the non-active group (*p* < 0.001). There were no significant differences between active and non-active participants on gender (*p* = 0.706) and number of kidney transplants, with the majority being first time KTRs (range 1–5) (*p* = 0.782).

**TABLE 1:** Participant characteristics (active vs. non-active users).

	Non-active users (n = 16)	Active users (n = 162)	*p*-value
Age: median (range)	67 (46–79)	55 (20–82)	<0.001
Gender: n female (%)	7 (43.8)	63 (38.9)	0.706
Graft functioning Delayed Primary non-function Unknown^a^	8 4 3 1	133 26 1 2	
Number of transplants: (range)	1 (1–5)	1 (1–3)	0.782
Donor Type: Living	2/16	85/162	<0.001

^a^ Unknown due to patient being from another center.

As the non-active users had initially registered, we included this group in the subsequent analyses according to the intention to treat principle. With regard to continuation, among the active and non-active users (n = 178), 135 participants were still in the program at the end of the study period, average of time in program of 198 days (range 1–508 days). This is an 76% retention rate. Of the 27 who stopped home-monitoring, the majority (n = 18) were transferred back to their referring regional hospital (on average 290 ± 71 days after start of home-monitoring program). Of the remaining 9: 5 stopped due to technical difficulties, 2 stopped without giving reason and 2 participants died. The overall dropout rate in our study was 13% (24/178) (no measurements & program stopped other than completion of program).

### Protocol Adherence

Protocol adherence per parameter (after the run-in period during admission) is presented in Table 2. At the date of data extraction, 6/162 participants were still in the run-in period and were not included in the analysis of protocol adherence. Participants were most adherent to measuring temperature (76.3% n = 119/156), followed by blood pressure measurements (75.6% n = 118/156). Participants were least adherent to protocol for the survey on smoking (156/419, 7.1%, n = 87/154), followed by medication taking (249/390, 34.2%, n = 52/152), subjective evaluations (171/263, 41.7% n = 48/115), the median adherence was 67% which corresponds to 1 evaluation and sex (138/205, 49.6%, n = 83/129).

**TABLE 2:** Overall protocol adherence per parameter (n = 156)^a^

Parameter	Total number of measurements recorded	Protocolled number of measurements^b^	Percentage of participants meeting criteria for protocol adherence^c^
Blood pressure	15,315	10,293	75.6% (118/156)
Heart rate	12,051	10,293	61.5% (96/156)
Weight	8,044	7,016	61.5% (96/156)
Temperature	9,681	4,904	76.3% (119/156)
Fluid intake	3,482	3,004	53.5% (81/156)
Urine production	3,305	3,004	51.9% (87/156)
Oxygen saturation	4,654	2,850	58.2% (85/146)
Pain score	1,842	2,850	37.7% (55/146)
Steps	1,788	950	33.6% (49/115)
Wound	1,585	950	53.4% (78/146)
Smoking	156	419	7.1% (11/154)
Medication problems	249	390	34.2% (52/152)
Subjective evaluation	171	263	41.7% (48/115)
Sex	138	205	49.6% (64/129)
Problems with defecation	90	98	61.5% (56/91)
Other (glucose)	4,261 (3,399)	0	-
**Total**	**66,812**	**47,489**	**22.4%**

Legend.

^a^ Patients (n = 6) still in the run-in period not included in this analysis.

^b^ Total number of protocolled measurements required taking into account the number of weeks in the program per participant.

^c^ Adherence protocol calculated based on >75% of measurements as stipulated by the protocol, summed over the total group of participants on the date of data extraction (31-12–2022).

Bold values are to highlight total measurements performed, required and protocol measurment adherence.

**FIGURE 4:**
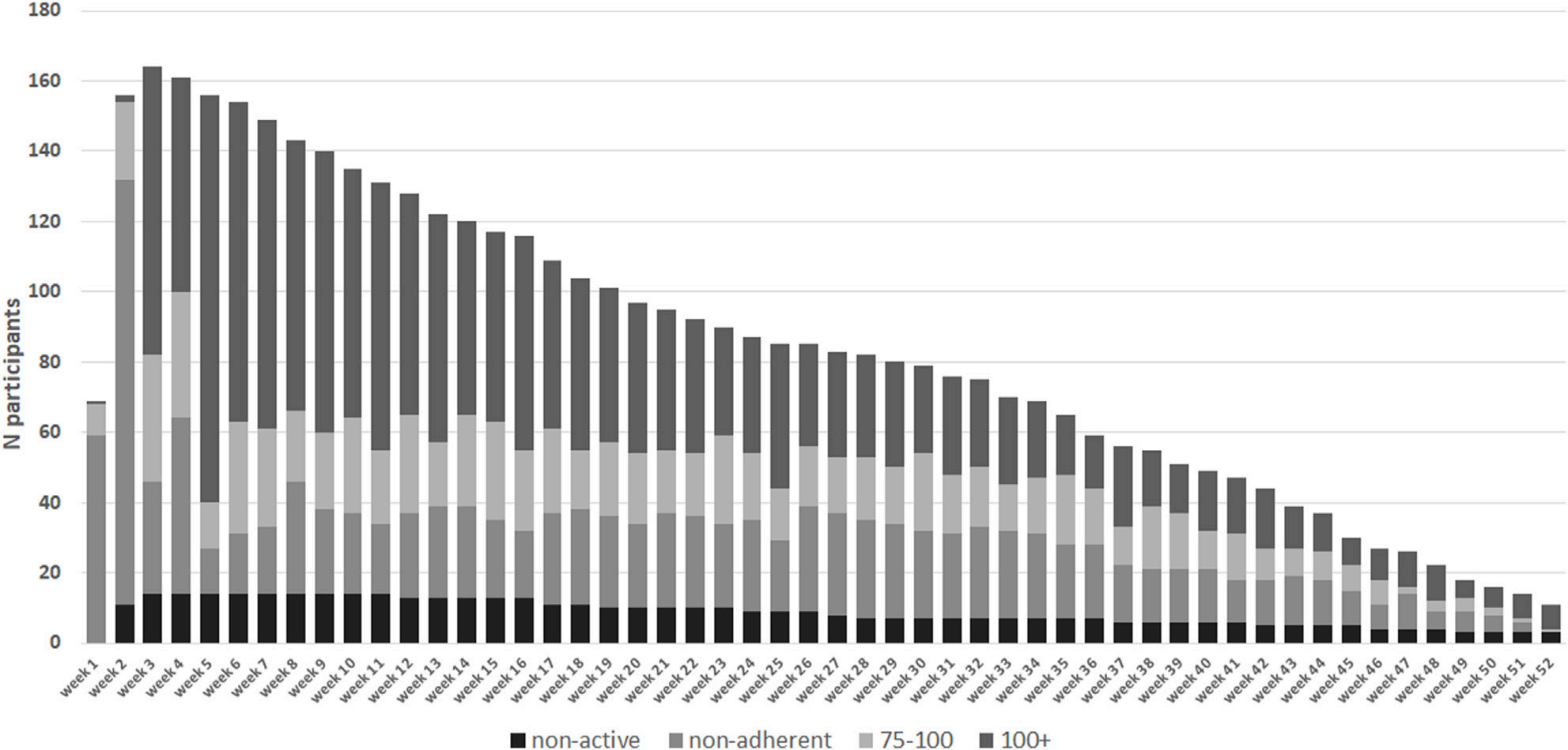
Absolute number of participants in each protocol adherence group (n = 178). Adherence per week from day of discharge from hospital. In week 1 and 2 data collection commences on day of discharge. Measurements collected during hospital admission in week 1 and 2 have been excluded. Black dashed bars represent the non-active group who have had not activated their home-monitoring app or entered any measurements.

Figure 4 shows the number of participants achieving protocol adherence divided into non-adherent (<75%), adherent (≥75–100%), and over adherent (≥100%), which also starts after the run-in period of the hospitalization period. Throughout the study there was a group of participants who were >100% adherent entering more measurements than stipulated according to protocol. In the 5th week, the intensity of the protocol decreases. At week 5, 156 participants were still in the sample. Of these, 13 were non-adherent, 13 were adherent, and 116 were over adherent (14 were non-active). This resulted in an overall adherence rate of 83% at week 5. At week 10 there were 135 participants, of which 23 were non-adherent, 27 participants were adherent, and 71 were over adherent and, (14 were non-active). This results in an overall protocol adherence rate of 73% at week 10. At week 20 there were 97 participants, of which 24 were non-adherent, 20 were adherent, 43 were over adherent (10 were non-active). This resulted in an overall protocol adherence rate of 66% at week 20. At week 40 there were 49 participants, 15 were non-adherent, 11 were adherent, 17 were over adherent (6 were non-active). This resulted in an overall protocol adherence rate of 57% at week 20. At the end of the retrospective study period 22.4% of all participants had an overall adherence to the protocol with all measurement types (Table 2).

### Subjective Evaluations

**FIGURE 5:**
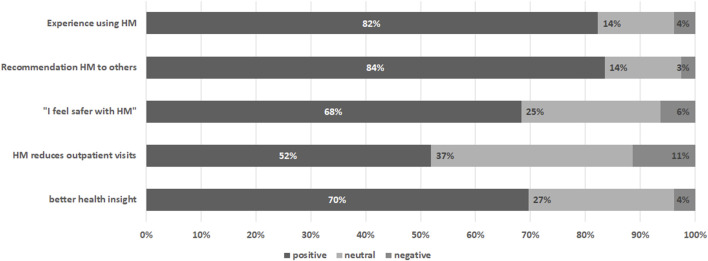
Subjective evaluation of the home-monitoring system per item. Percentages are based on the average score of the participant rounded to nearest likert 5-scale value.

Questions on subjective evaluation of the home-monitoring system were completed by 79 individuals (69% of individuals prompted n = 79/115, 44% of total group n = 79/178), of whom 45 answered this questionnaire multiple times (n = 171 evaluations). On the first item measuring overall experience of the home-monitoring system responses were positive, with on average a score >4 on a 5-point scale (4.19 ± 0.86) (see Table 3). Also participants generally agreed that they felt safer with home-monitoring (3.81 ± 0.95), that it reduced visits to the hospital (3.56 ± 1.4) and it gave better insight into health (3.77 ± 0.87). Participants were highly likely to give recommendations’ of the system to others (8.15 ± 2.19), see Figure 5.

**TABLE 3:** Subjective evaluation average scores and change over time.

Items	Average overall (n = 79)	First entry average (n = 45)	Last entry average (n = 45)	Delta (n = 45)	*p*-value
*Experience using home-monitoring*	4.19 ± 0.86	4.16 ± 0.90	4.2 ± 0.94	0.04	0.321
*“I feel safer with home-monitoring"*	3.81 ± 0.95	3.82 ± 0.96	3.98 ± 0.92	0.16	0.127
*HM reduces outpatient visits*	3.56 ± 1.04	3.44 ± 0.99	3.56 ± 1.20	0.12	0.221
*Better health insight*	3.77 ± 0.87	3.84 ± 0.80	3.73 ± 0.92	−0.09	0.241
*Recommendation home-monitoring to others*	8.15 ± 2.19	7.38 ± 2.60	8.27 ± 2.35	0.66	0.010*

A subgroup analysis was conducted among those with multiple subjective evaluation measurements (see Table 3). Paired T-test between start and last measurement showed significant increase score in time with “recommendations” (*p* = 0.010). There was no significant change over time in subjective evaluation items “experience” (*p* = 0.321), “safety” (*p* = 0.127), “outpatient visits” (*p* = 0.221) and “insight” (*p* = 0.241).

**FIGURE 6:**
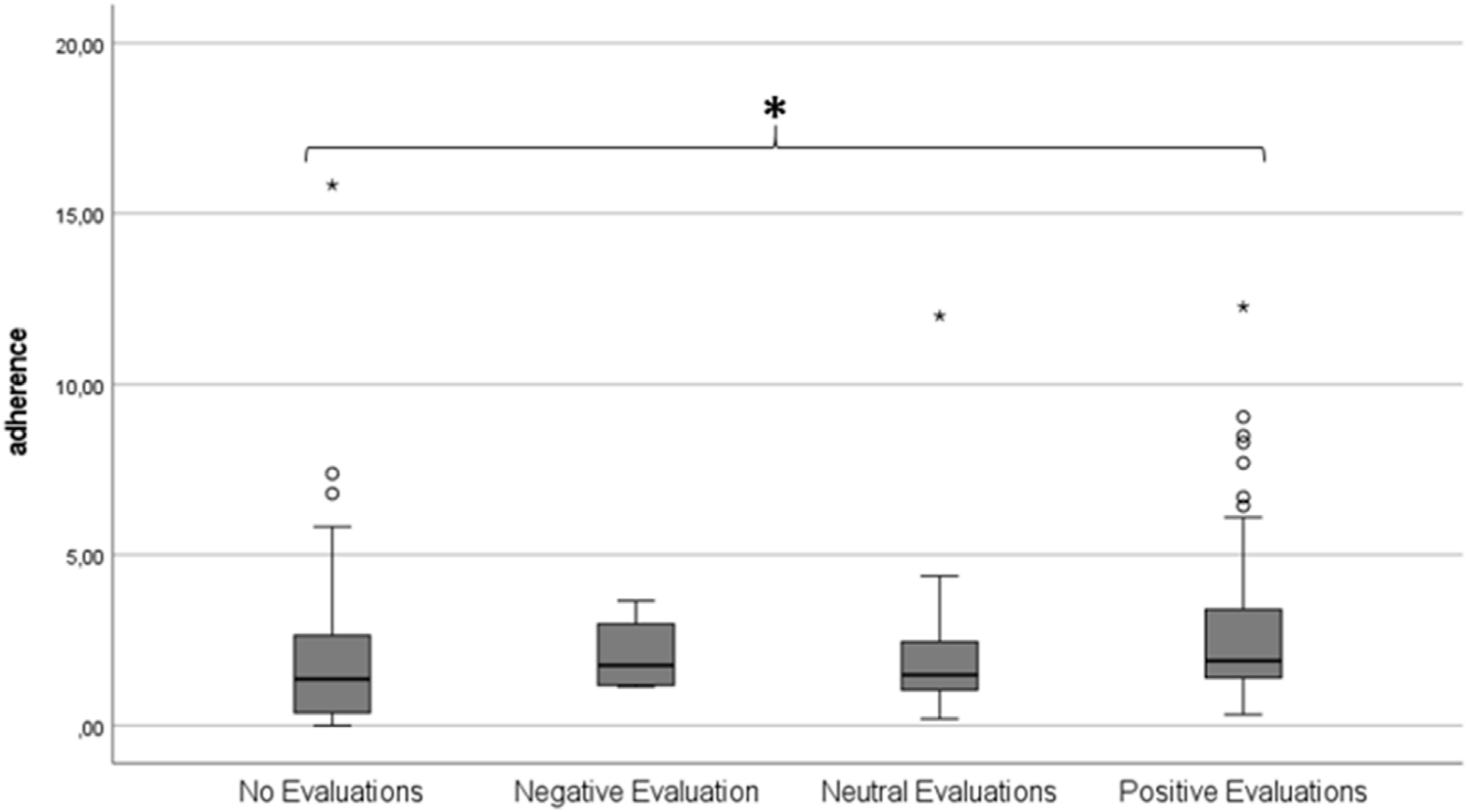
Boxplot showing total average protocol adherence according to subjective evaluation group. A score of 1.00 represents 100% protocol adherence.

Finally, total protocol adherence was compared between participants who differed in their subjective evaluation of the home-monitoring (see Figure 6). Four groups were defined based on average subjective evaluation scores: no evaluations (n = 82), negative evaluation (n = 4), neutral evaluation (n = 16), positive evaluation (n = 60). One-way ANOVA did not show any differences between groups in protocol adherence (*p* = 0.25).

## Discussion

After implementing home-monitoring as standard care in our center, these findings show that KTRs are more than ready to adopt the new technology. Out of the 297 KTRs, 256 had registered for one of the home-monitoring packages. The 178 KTRs who started the “*de novo*” home monitoring program showed high retention to the protocol with 76% still in use at our cutoff date. After the run-in period and discharge from hospital, protocol adherence was high for the majority of participants although this tapered off over time. There was a subgroup of participants that were more than 100% protocol adherent throughout in total measurements. Lastly, subjective evaluations were carried out by 69% of participants who made it in the protocol, and were generally positive. A positive evaluation does not appear to be related to protocol adherence, however, those who do not complete a subjective evaluation were on average less adherent to the protocol.

High uptake of home-monitoring has been reported previously [3]. Interestingly in our study, we also found high uptake among older transplant recipients. In the Netherlands, elderly are more reluctant in using eHealth based smartphone apps compared to younger adults [16]. However, in this specific group of KTRs we were able to include the entire spectrum of ages. In general, reluctance to using the home-monitoring was low, given that only 8% (16/178) were non-active users. This is low compared to a study in Berlin by Duettmann that reported 19% refusal from participation. We note however, that non-active users, that is those who initially agreed to use the home-monitoring system but at a later date did not proceed, were more likely to be older. Although reasons of not using the app were not recorded, it is possible that this sub-group require more assistance or support to use the system. Greater insight into reasons for refusal as well as barriers to activation would offer targets for improving the system, and specifically whether support needs to differ according to age.

For those who were offered the program and actively home-monitored, continuation was high. The overall dropout rate in our study was 13%, resulting in a continuation rate that was comparable or higher than other studies. For example, a study in Berlin showed a similar dropout rate of 6% (8/139) [17]. Whereas a study in Seoul reported high dropout rates of 53% after 1 month [18], however, their sample was enrolled after transplantation in a randomized control trial and not part of standard care which may explain the higher drop-out rate. We also note that participation in our home-monitoring system is typically up to 12 months when KTRs are transferred back to their original regional hospital for further follow-up, where we were not able to measure continued use on the long-term.

Protocol adherence is an important indicator of whether the protocol developed by professionals is acceptable and achievable for recipients. In this study, after the run-in period and discharge from the hospital, protocol adherence was high when protocol measurements were lower. Vitals such as blood pressure, heart rate, weight and temperature were measured more than the protocol suggested, which shows interest and/or dedication by KTRs participants. Also, certain topics measured by questionnaires, such as smoking and sex, were completed on different times than the protocol stipulated compared to other topics such as urine production, liquid intake or medication. Some topics may be more important to patients than others. From a clinical perspective, the information generated from the questionnaires on accurate medication taking are important but protocol adherence for these were relatively low. There may be a lack of awareness as to the reason for repeated administration. For some questionnaires it is possible that questionnaire burden reduced the rate of completion. Some topics (such as sex or taking medication) may be more sensitive and KTRs may be reluctant to submit answers on them through an app. Further research is needed to understand how these psychological factors may influence engagement with the home-monitoring system.

Our retrospective study highlights an important consideration for professionals developing home-monitoring protocols not over-burden users while still obtaining enough information to allow effective monitoring. In the first 2 weeks, recently discharged participants had low adherence and overall within the protocol only 22.4% were adherent to the entire protocol during the entire study period. Participants were less protocol adherent to certain parameters than others. To make improvements it will be important to understand why protocol adherence differs across parameters. Furthermore, previous studies suggest that active involvement of health professionals (e.g., discussing results) and reminders positively influence adherence to measurements and medication [4, 19, 20]. In the home-monitoring system in this study, it is likely that reminders sent through the app promoted protocol adherence. Whilst these notifications can be turned off, what the setting was with our participants was not clear. Notification settings could be linked with non-adherence/adherence as one factor for dropout rate and adherence. The long-term engagement and retaining of transplant recipients in home-monitoring programs is paramount for sustained benefits. This study identifies the great potential of home-monitoring as standard care for KTRs and other organ recipients. Implementing home-monitoring as standard care is likely to have contributed to the high level of uptake.

Introduction of the technology and instruction on use during hospital admission for transplantation may help remove barriers to use as recipients can ask questions and ask for guidance when needed before discharge. Starting during hospitalization with entering measurements could provide routines for patients which could be linked to the high number of records on heartrate, temperature and blood pressure. When considering development of a home-monitoring protocol, professionals should involve recipients to help assess feasibility and limit undue burden as an unduly intensive schedule may subsequently have a negative influence on protocol adherence as seen in our center early on with high intensity measurement protocol in the first month. We note that there were active users that had a protocol adherence level above 100%. The potential reasons for and consequences of this are not yet clear, a possibility could be due influence from the health professional, suggesting alternative protocols or caution. Another is the routine learned/acquired in the hospital/early discharge.

Patient attitude towards home-monitoring were positive, evidenced by active participation in large number of recorded measurements and overall positive subjective evaluations. The positive evaluations are in line with other studies implementing home-monitoring modalities [2, 17, 21]. A shortcoming in our study was that we were not able to capture the perspectives of those who did not engage with the home-monitoring system.

Despite the positive results, further improvements are still possible to enhance implementation of home-monitoring the facilitators and barriers experienced of KTRs and home-monitoring. For future research on how to improve the system a number of target groups can be identified, for example, those who do not engage with home-monitoring, those who monitor on paper, and those who are more than 100% adherent. It is not known if some users become overly involved or compulsive about monitoring and what kind of effect this may have on quality of life. In addition to KTRs, investigation of the perspectives of transplant professionals would be help further development of the system and improve on the barriers that they might see themselves in the integration of home-monitoring for KTRs care. In the future it will be also important to investigate the level of engagement by professionals with the home-monitoring system and their influence on recipient behavior. Qualitative research that explores attitudes and acceptance, perceived benefits for both patients and the healthcare system, and willingness to discuss data and perceived barriers to implementation would be informative.

In our center this home-monitoring has now been implemented as standard care, but we will continue to make improvements, which may also lead to (further) changes in the roles and responsibilities of patient and transplant professionals [22]. Developing the home-monitoring system into a more autonomous self-managed care approach is a promising avenue in which monitoring protocols are (better) tailored to individual patient needs, medical histories, and (known) risk profiles. In the future, home-monitoring may go one step further by reducing the role of professionals by “closing the loop.” In a closed loop home-monitoring system patients carry out blood assays at home for medication dosing which is adjusted based on algorithms that can take many clinical factors into account. Moreover, it may be possible to combine data from home-monitoring with other data sources such as patient-reported outcomes, quality of life assessments, and long-term clinical outcomes through integrated dashboards and interactive feedback. This approach will contribute to a more comprehensive understanding of recipient health and wellbeing.

In conclusion, this study demonstrated the feasibility of implementing home-monitoring as standard care after kidney transplantation. We found high uptake, high protocol adherence and the continued use of home-monitoring among KTRs, with positive subjective evaluations and recommendation of the system to others. Areas for further investigation and improvement of the system were identified.

## Data Availability

The raw data supporting the conclusions of this article will be made available by the authors, without undue reservation.
