# Supplementary material for: The SElf-Care After REnal Transplantation Study: A Retrospective Evaluation of a Home-Monitoring Program Implemented as Standard Care
Source: Transpl Int. 2024 Jul 22;37:13192. doi: 10.3389/ti.2024.13192 (PMC11298348; doi:10.3389/ti.2024.13192)
Supplement: Supplementary file 1 [file DataSheet1.docx]

**SUPPLEMENTARY MATERIAL**

Protocol

During the hospital stay:

• Pain score 3 times a day, weigh once a day (in the morning), measure blood pressure 3 times a day, check heart rate 3 times a day, measure temperature 3 times a day, measure oxygen levels 3 times a day, measure urine output and fluid intake once a day, check the wound once a day.

• We encourage you to move as much as possible, and you can also track your steps once a day.

After discharge from the hospital:

• First four weeks: weigh once a day, measure blood pressure twice a day, measure temperature twice a day, measure urine output and fluid intake once a day, check the wound once a day. • Week 5-6: weigh once a day, measure blood pressure twice a week (in the morning and evening), measure temperature twice a week (in the morning and evening), use a step counter twice a week.

• Week 7-12: weigh once a week, measure blood pressure twice a week (in the morning and evening), use pedometer twice a week.

After this period, you will gradually decrease the frequency of measurements, but remember that it's always okay to measure more often if you prefer!

• Month 3-6: weigh once a week, measure blood pressure once a week (in the morning and evening), use pedometer once a week.

• Month 7-8: weigh once every 2 weeks, measure blood pressure once every 2 weeks (in the morning and evening), use pedometer once a week.

• From month 9 onwards: weigh once a month, measure blood pressure once a month (in the morning and evening), use pedometer once a week.
